# Biomarkers of Redox Balance Adjusted to Exercise Intensity as a Useful Tool to Identify Patients at Risk of Muscle Disease through Exercise Test

**DOI:** 10.3390/nu14091886

**Published:** 2022-04-29

**Authors:** Pierre-Edouard Grillet, Stéphanie Badiou, Karen Lambert, Thibault Sutra, Maëlle Plawecki, Eric Raynaud de Mauverger, Jean-Frédéric Brun, Jacques Mercier, Fares Gouzi, Jean-Paul Cristol

**Affiliations:** 1 PhyMedExp, INSERM U1046, CNRS UMR 9214, University of Montpellier, CHU Montpellier, 34295 Montpellier, France; pe-grillet@chu-montpellier.fr (P.-E.G.); s-badiou@chu-montpellier.fr (S.B.); karen.lambert-cordillac@umontpellier.fr (K.L.); t-sutra@chu-montpellier.fr (T.S.); m-plawecki@chu-montpellier.fr (M.P.); eric.raynaud-de-mauverger@chu-montpellier.fr (E.R.d.M.); jf-brun@chu-montpellier.fr (J.-F.B.); j-mercier@chu-montpellier.fr (J.M.); jp-cristol@chu-montpellier.fr (J.-P.C.); 2 Department of Biochemistry and Hormonology, CHU Montpellier, 34295 Montpellier, France; 3 Department of Physiology, University of Montpellier, CHU Montpellier, 34295 Montpellier, France

**Keywords:** metabolomics, Liquid Chromatography tandem Mass Spectrometry (LC-MS/MS), cardiopulmonary exercise test (CPET), tricarboxylic citric acid cycle, exercise intolerance, myopathy, maximal oxygen uptake

## Abstract

The screening of skeletal muscle diseases constitutes an unresolved challenge. Currently, exercise tests or plasmatic tests alone have shown limited performance in the screening of subjects with an increased risk of muscle oxidative metabolism impairment. Intensity-adjusted energy substrate levels of lactate (La), pyruvate (Pyr), β-hydroxybutyrate (BOH) and acetoacetate (AA) during a cardiopulmonary exercise test (CPET) could constitute alternative valid biomarkers to select “at-risk” patients, requiring the gold-standard diagnosis procedure through muscle biopsy. Thus, we aimed to test: (1) the validity of the V’O_2_-adjusted La, Pyr, BOH and AA during a CPET for the assessment of the muscle oxidative metabolism (exercise and mitochondrial respiration parameters); and (2) the discriminative value of the V’O_2_-adjusted energy and redox markers, as well as five other V’O_2_-adjusted TCA cycle-related metabolites, between healthy subjects, subjects with muscle complaints and muscle disease patients. Two hundred and thirty subjects with muscle complaints without diagnosis, nine patients with a diagnosed muscle disease and ten healthy subjects performed a CPET with blood assessments at rest, at the estimated 1st ventilatory threshold and at the maximal intensity. Twelve subjects with muscle complaints presenting a severe alteration of their profile underwent a muscle biopsy. The V’O_2_-adjusted plasma levels of La, Pyr, BOH and AA, and their respective ratios showed significant correlations with functional and muscle fiber mitochondrial respiration parameters. Differences in exercise V’O_2_-adjusted La/Pyr, BOH, AA and BOH/AA were observed between healthy subjects, subjects with muscle complaints without diagnosis and muscle disease patients. The energy substrate and redox blood profile of complaining subjects with severe exercise intolerance matched the blood profile of muscle disease patients. Adding five tricarboxylic acid cycle intermediates did not improve the discriminative value of the intensity-adjusted energy and redox markers. The V’O_2_-adjusted La, Pyr, BOH, AA and their respective ratios constitute valid muscle biomarkers that reveal similar blunted adaptations in muscle disease patients and in subjects with muscle complaints and severe exercise intolerance. A targeted metabolomic approach to improve the screening of “at-risk” patients is discussed.

## 1. Introduction

The skeletal muscle diseases encompass a large and heterogeneous spectrum of inherited or acquired muscle diseases (immobility, inflammatory, myopathies (inherited, metabolic…), intensive care unit-acquired weakness, and chronic systemic diseases [1,2]) characterized by a decreased contracting ability and/or a muscle mass alteration. Metabolic myopathies are characterized by an impairment of the muscle oxidative metabolism, in relation with an altered coenzyme or substrate availability (tricarboxylic acid (TCA) cycle β-oxidation substrates, ketone bodies or amino-acids), or in relation with an impaired substrate oxidation (mitochondrial oxidative phosphorylation (OXPHOS) deficiency) [3]. Yet, the oxidative metabolism impairment is not limited to muscle metabolic myopathies and is also widely observed in muscle diseases, as well as in deconditioning [4], ageing [5] and chronic diseases. Clinically, a muscle oxidative metabolism impairment limits the muscle ATP supply, alters some metabolite concentrations, which leads to a cellular redox disequilibrium, fiber damages and work efficiency/endurance impairment [4]. Thus, the clinical manifestations cover a broad range of resting and exercise symptoms (myalgia, cramps, exercise intolerance, dyspnea, weariness…) [1]. Because these symptoms are not specific, the diagnosis of a muscle oxidative metabolism impairment requires functional and/or biological investigations [6].

The gold-standard method for the diagnosis of a muscle oxidative metabolism impairment is muscle biopsy, combined with biochemical, enzymatic analyses and oxygraphic mitochondrial function assessment [7,8,9]. However, it is an invasive procedure that requires specialized and cost-expensive expertise [7,8]. Thus, non-invasive tests are warranted to better screen “at-risk” patients indicated for a muscle biopsy. Among potential non-invasive biomarkers [10], the cardiopulmonary exercise testing (CPET) provides indexes of exercise capacity (maximal O_2_ uptake (V’O_2max_) and work rate (W_max_) [11,12,13]) that are correlated with muscle TCA enzymes [14] and maximal oxygen consumption of the muscle mitochondria (V_max_) [15]. Other submaximal indexes like the V’O_2_ at the first ventilatory threshold (V_T_), and the V’O_2_/work rate slope have also been related with muscle impairment [11,15]. While these CPET biomarkers were discriminative, they were not sensitive enough [16].

Among other non-invasive approaches, plasma circulating biomarkers such as muscle energy substrates (lactate, TCA cycle intermediates and ketone bodies), that increase during exercise in the plasma [4,17], also constitute interesting candidates. Given the impairment in substrate availability or OXPHOS in muscle diseases, plasma energy substrate kinetics during a maximal exercise test (CPET) should differ between healthy subjects and muscle disease patients as it changes with the training status [18]. Yet, a recent study in mitochondrial myopathy did not find any abnormal blood energy substrate (lactate (La) and pyruvate (Pyr)) at peak exercise [19]. A similar lack of difference in ketone bodies (β-hydroxybutyrate (BOH) and acetoacetate (AA)) has also been reported in these patients [20]. However, given that these substrates are correlated with the exercise intensity and subject’s capacity [17,21], adjusting the blood substrate level to the V’O_2_ at which the sampling is performed should discriminate mitochondrial myopathy patients from healthy subjects. In addition, La/Pyr and BOH/AA ratios constitute valid markers of the cytoplasm and mitochondrial redox status, respectively [22]. These ratios are increased by muscle acidosis and ROS production [4] during exercise [23], and could be higher when adjusted to the exercise intensity in mitochondrial myopathy patients compared to healthy controls [24].

To date, the validity of V’O_2_-adjusted energy and redox biomarkers during a CPET has never been tested compared to muscle function indexes (V’O_2max_ and fiber mitochondrial respiration). Validation of such biomarkers during a CPET could allow to discriminate healthy subjects from subjects with muscle complaints or muscle disease. Recently, metabolomics methods have provided a global view of the muscle metabolic changes during exercise [21,25]. Therefore, assessing simultaneously a full panel of five metabolites around the TCA cycle during exercise could further improve the discriminating capacity of the test.

Altogether, to test the validity of the V’O_2_-adjusted La, Pyr, BOH and AA during a CPET for the assessment of the muscle oxidative metabolism, we aimed to test correlations between these V’O_2_-adjusted La, Pyr, BOH and AA and the muscle functional parameters during exercise and with the mitochondrial respiration parameters in healthy and muscle complaints subjects. In addition, we compared the V’O_2_-adjusted energy and redox markers between healthy subjects, complaints subjects and muscle disease patients. Last, the discriminative value of a full panel of eight V’O_2_-adjusted TCA cycle-related metabolites was further explored in healthy vs. muscle complaints subjects.

## 2. Materials and Methods

### 2.1. Population

We conducted a retrospective study from an open cohort of subjects included from September 2019 to December 2021. The Institutional Review Board of Montpellier University Hospital approved the study (IRB-MTP_2022_03_202201039), and informed consent was obtained from each patient. All the subjects and patients had a medical examination performed by experienced physicians of the clinical physiology department of the Montpellier hospital. The inclusion criteria were: (1) adults aged from 18 to 85 years old; (2) referred to the clinical physiology department of the Montpellier University Hospital, France, by physicians for exploration of muscle complaints at rest or during exercise (myalgia, cramps, fatigue, breathlessness) or exercise intolerance; (3) no medical treatment impacting the exercise or muscle capacity; (4) no contra-indication to a maximal CPET. For subgroup analysis, patients with muscle complaints were classified according to their exercise capacity [26] as patients with severe exercise intolerance (<65% of predicted V’O_2max_), mild exercise intolerance (between 65 and 85% of predicted V’O_2max_) and with normal exercise capacity (>85% of predicted V’O_2max_). A muscle biopsy was performed in complaints subjects whose exploration revealed severe alterations of exercise capacity or biological abnormalities. Healthy subjects were included in a control group if (1) aged from 18 to 85 years old; (2) free from muscle symptoms, exercise limitation, or medical disease; and (3) no medical treatment. Last, patients with a diagnosis of myopathy (based on a muscle histological analysis or genetic and/or neurological criteria) constituted the muscle disease group (*n* = 9): one patient with McArdle’s disease, three patients with mitochondrial cytopathy diagnosed on a muscle biopsy, one patient with undefined myopathy with altered biopsy suggesting myogenic myopathic syndrome, one patient with type III glycogenosis, one patient with MELAS syndrome, one patient with ANO5 mutation, and one patient with myopathy linked to ND5 mutation.

### 2.2. Assessments

After a medical examination, anthropometric parameters were assessed (weight; height). Then, all subjects and patients underwent a segmental multifrequency bioelectronic impedance meter analysis to assess the body composition [27] with an impedance plethysmograph (Biacorpus RX4000 software, BodyComp 8.4, Medi-Cal Healthcare GmbH, Karlsruhe, Germany). Body fat mass (FM) and fat-free mass (FFM) were calculated for each segment of the body, according to the manufacturer’s database-derived disclosed equations, and total water with published equations using the classical cylindrical model and the Hanai mixture theory [28]. The FM, FFM, and muscular mass were expressed in kilograms and as a percentage of total body mass. Muscle mass index was calculated as muscular mass/height^2^ and expressed in kg/m^2^. Then, a venous 32-mm catheter was placed into a superficial antecubital forearm vein by a nurse.

### 2.3. Cardiopulmonary Exercise Test

After at least 3 h of fasting, the participants performed a CPET on an electromagnetically braked cycle ergometer (Ergoline Bosch 500, Ergoline, Bitz, Germany) connected to a breath-by-breath device (COSMED Quark cardiopulmonary exercise testing, COSMED, Rome, Italy) for oxygen consumption (V’O_2_) and carbon dioxide production (V’CO_2_) measurements. The theoretical maximal oxygen output (V’O_2max_) was calculated for all patients using Wasserman equations [29]. The test was performed according to international standards. Briefly, after a 3-min constant load warm-up corresponding to 20% of the targeted maximal workload, the load was progressively increased every minute by 10% of the targeted maximal workload, until patient’s exhaustion. During the exercise test, heart rate, ECG, blood pressure, and transcutaneous oxygen saturation were monitored. In addition, the subject’s muscle weariness was recorded every minute using an EVA scale. Maximality criteria had to match the international guidelines. Maximal power output was the maximal workload sustainable, and V’O_2max_ was the mean value during the last 20 s of the test. The 1st ventilatory threshold was identified by the physician supervising the CPET, according to the following criteria: increase in total ventilation, increase in respiratory exchange ratio, increase in patient’s weariness and the theoretical range of V’O_2_ at V_T1_ (30–60% of predicted V’O_2max_) as recommended [30].

### 2.4. Samples

Biological samples were collected using the catheter at three different time points: at rest, at the estimated 1st ventilatory threshold (eV_T1_) and at the acme of the effort. The blood was collected in Vacutainer^®^ heparinized tubes and immediately deproteinized in parallel with perchloric acid 1N (ITW Reagents, Darmstadt, Germany) (1:1) for routine redox marker determination and with trichloroacetic acid 10% (VWR Chemicals, Radnor, PA, USA) (1:1) for TCA-related metabolite determination. After vortexing, samples were then centrifuged (2000× *g*, 10 min, +4 °C) and supernatant was stored at −80°C until analysis.

### 2.5. Energy and Redox Biomarkers

After neutralization of the supernatant, La, Pyr, BOH and AA were routinely determined on an open automated analyzer (BS-480, Mindray, Shenzhen, China) with commercial enzymatic methods using lactate dehydrogenase, glutamate pyruvate transaminase and β-hydroxybutyrate dehydrogenase, respectively (Biosentec, Portet-sur-Garonne, France).

### 2.6. TCA Cycle-Related Metabolites

Exploration of TCA cycle metabolites in addition to La, Pyr and ketone bodies was performed using liquid chromatography—mass spectrometry (LC-MS/MS) in all controls and in 39 subjects with muscle complaints. Analyses were performed on an Acquity UPLC system (Waters Corporation, Milford, MA, USA) coupled to a mass spectrometer XEVO TQD (Waters Corporation). The chromatographic separation was achieved on an Acquity Premier CSH Phenyl-Hexyl column (2.1 × 100 mm, 1.7 μm, Waters Corporation). Mobile phase A consisted of 0.1% formic acid in water, while mobile phase B consisted of 0.1% formic acid in acetonitrile. The column flow rate was set at 400 µL/min and the column temperature was maintained at 50 °C with an injection of 10 µL and a total run analysis of 11 min for simultaneous determination of La, Pyr, BOHB, citrate, malate, succinate, fumarate and alpha-ketoglutarate.

### 2.7. Skeletal Muscle Biopsy

Vastus lateralis muscle biopsies were performed using the percutaneous Bergström technique after local anesthesia (Xylocaine). No biopsy-related complications were further reported. Muscle samples were then split into two portions: one portion was placed in a standard medium for histoenzymological analysis, the other one was immediately placed in an ice-cold relaxing solution (at ionic strength 160 (potassium methanesulfonate), pH 7.1 containing (in mM) 10 EGTA-calcium buffer (free Ca^2+^), 20 imidazole, 3 KH2PO4, 1 MgCl2, 20 taurine, 0.5 DTT, 5 MgATP, and 15 phosphocreatinefor in situ respiration studies. The fiber bundles were separated with sharp-ended needles, leaving only small areas of contact, and were incubated in 1 mL of the above solution (4 °C) containing 50 µg/mL saponin for 30 min under continuous stirring. To completely remove saponin, the fibers were washed under continuous stirring with relaxing solution for 10 min (4 °C); to remove free ATP, the fibers were then washed with oxygraph solution for 2 × 5 min (4 °C) using the relaxing solution, except that MgATP and phosphocreatine were replaced by 2 mM malate, 3 mM phosphate, and 2 mM fatty acid-free bovine serum albumin (pH 7.1). After washing, the fibers were kept on ice in oxygraph solution until determination of mitochondrial respiration activity.

### 2.8. Mitochondrial Respiration

The respiratory parameters of the total mitochondrial population were studied in situ, as previously described [18,31], using a Clark electrode (Strathkelvin Instruments, Glasgow, UK). Measurements were carried out at 30 °C under continuous stirring in 3 mL of oxygraph solution with different respiratory substrates (in mM): either 5 glutamate + 2 malate or 10 pyruvate + 2 malate. For each sample, basal oxygen consumption without ADP was first recorded (V_0_), and then the ADP-stimulated maximal respiration (V_max_) was determined in the presence of a saturating concentration of ADP (2 mM). At the end of the measurement, the cytochrome c test was used to investigate the outer mitochondrial membrane status. After the following respiratory measurements, the fiber bundles were removed, dried overnight, and weighed the day later. Respiration rates (V_max_) were expressed in micromoles of O_2_ per minute per milligram of dry weight (μmolO_2_/min/mg). V_max_ with glutamate and V_max_ pyruvate revealed complex I, III and IV activities.

### 2.9. Statistical Analysis

According to their distribution, baseline clinical and functional parameters were provided as mean ± SD and biochemical parameters were provided as mean ± SEM or median (interquartile range 25–75%). Because energy substrate, redox markers and TCA cycle intermediates are correlated with exercise intensity, these markers were adjusted to exercise intensity, and thus reported to the V’O_2_ measured at the moment of the blood sample (expressed in % of the predicted V’O_2max_). Univariate correlations between these V’O_2_-adjusted biomarkers and clinical, functional and mitochondrial respiration parameters were tested using Pearson’s correlation coefficients. Stepwise multivariate regression analysis was performed using V’O_2max_ as the dependent variable and included the significantly correlated variables as independent variables. Mallow’s Cp was used as specific selection criteria to find the best model from all possible combinations. The best model would have a Mallow’s Cp variable equal to the number of variables in the model [32]. R^2^ and Mallow’s Cp were calculated using XLStat (Addinsoft, New York, NY, USA). The V’O_2_-adjusted biomarkers and other clinical and functional parameters were compared between groups at baseline using a one-way analysis of variance or the Kruskal–Wallis test, depending on the data normality. V’O_2_-adjusted biomarkers measured at all time points of the CPET were compared with a mixed-model effect using GraphPad Prism V9.0 (GraphPad Software, San Diego, CA, USA) including group effect (G), time effect (T), the interaction between these factors (G * T) as the fixed effect, and the subject effect as the random effect. Assumptions of normal distribution of the residuals and homoscedasticity were graphically verified for each mixed-model effect test. Post-hoc analyses were performed using Dunn’s test when Time*Group interaction was significant. Data were plotted with GraphPad Prism V9.0 (GraphPad Software, San Diego, CA, USA). A *p*-value of <0.05 was considered significant.

## 3. Results

**Figure 1:**
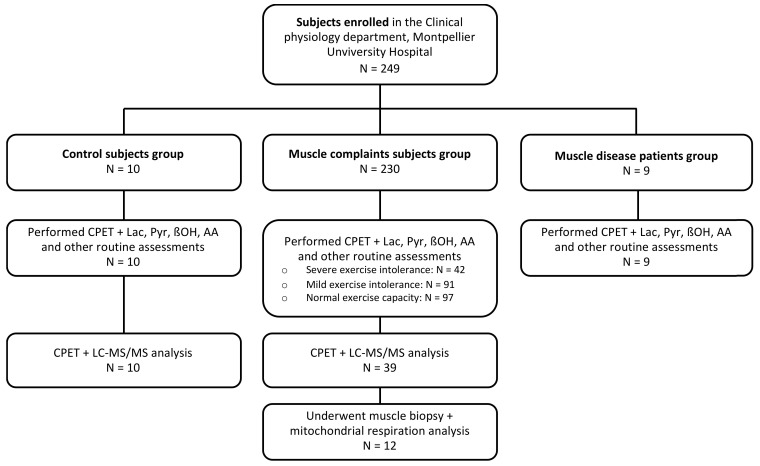
Flow-chart diagram of the studied subjects and assessments. AA: Acetoacetate; ßOH: beta-hydroxybutyrate; CPET: cardiopulmonary exercise test; Lac: lactate; LC-MS/MS: chromatography liquid tandem mass spectrometry; Pyr: pyruvate.

From September 2019 to December 2021, 230 consecutive subjects with muscle complaints without diagnosis, 9 patients with proved muscular disease, and 10 healthy control subjects underwent a CPET in the department of clinical physiology at the Montpellier University Hospital, France (Figure 1). The main complaints of the 230 subjects were myalgia, exercise-induced myalgia, fibromyalgia, exercise intolerance or elevated creatine kinase (CK) on laboratory tests. Age (44.9 ± 15.8 vs. 37.1 ± 13.3 y.o.; *p* = 0.126), female gender (51.3% vs. 50.0%; *p* = 1.00), and skeletal muscle index (8.8 ± 2.6 kg/m^2^ vs. 8.4 ± 1.4; *p* = 0.630) did not significantly differ between subjects with muscle complaints and healthy subjects. However, a reduction in aerobic exercise capacity, as evidenced by a reduction in V’O_2max_ (82.8 ± 23.6 vs. 100.7 ± 17.0% pred.; *p* < 0.05), but not V’O_2_@V_T1_ (57.0 ± 16.3 vs. 65.7 ± 17.4% of predicted V’O_2max_; *p* = 0.10) was observed in subjects with muscle complaints compared to healthy subjects.

### 3.1. Energy Substrate and Redox Markers Assessed during a Standardized CPET Are Associated with the Aerobic Exercise Capacity

In healthy as well as in muscle complaint subjects, significant univariate correlations between the four V’O_2_-adjusted biomarkers, lactate (La), pyruvate (Pyr), β-hydroxybutyrate (BOH), acetoacetate (AA), and muscle parameters obtained during CPET including weariness, V’O_2_@V_T1_ and V’O_2_/work rate slope, were observed (Table 1).

Including exercise functional parameters only, a model including weariness, V’O_2_@V_T1_ and V’O_2_/work rate slope explained 61% of the V’O_2max_ variability. Adding biological parameters (V’O_2_-adjusted TCA intermediates) to the muscle functional parameters during a standardized CPET only slightly improved the determination of the subject’s V’O_2max_ (Table 2). Adding BOH (both at V_T1_ and at maximal exercise) to the V’O_2_/work rate slope and V’O_2_@V_T1_ improved the V’O_2max_ adjusted R^2^ from 61 to 66%. Adding more than five variables was associated with decreased Mallow’s Cp under the number of variables included in the model (Table 2).

### 3.2. Energy and Redox Markers Assessed during a Standardized CPET Are Associated with Muscle Fiber Mitochondrial Respiration

In muscle complaint subjects requiring a muscular biopsy (*n* = 12, Supplementary Table S1), the mitochondrial respiration was significantly correlated with exercise muscle CPET parameters. V_max_ glutamate or V_max_ pyruvate were significantly correlated with V’O_2max_ (r = 0.739; *p* < 0.01 and r = 0.688; *p* < 0.05, respectively) and V’O_2_@V_T1_ (r = 0.640; *p* < 0.05 and r = 0.714; *p* < 0.001).

Regarding the V’O_2_-adjusted biomarkers (Supplementary Table S1), La@eV_T1_ and Pyr@eV_T1_ were inversely correlated with V_max_ pyruvate (r = −0.684; *p* < 0.001 and r = −0.649; *p* < 0.001, respectively). Regarding the V’O_2_-adjusted ketones bodies, BOH@rest, BOH@eV_T1_ and BOH@max were significantly correlated with V_max_ glutamate (r = −0.729; *p* < 0.001, r = −0.765; *p* < 0.001 and r = −0.748; *p* = 0.053, respectively), and AA@eV_T1_ and AA@max were significantly correlated with V_max_ glutamate (r = −0.608; *p* < 0.001 and r = −0.621; *p* < 0.001, respectively). Finally, BOH/AA ratio at rest, eV_T1_ and maximal exercise showed significant correlation with V_max_ glutamate (r = −0.744; *p* < 0.001, r = −0.688; *p* = 0.088 and r = −0.736; *p* = 0.059, respectively).

### 3.3. Energy and Redox Markers Assessed during a Standardized CPET Can Discriminate Subjects with Muscle Complaints and Patients with Muscle Disease from Healthy Subjects

Table 3 shows the differences in muscle CPET parameters in the three study groups.

**Figure 2:**
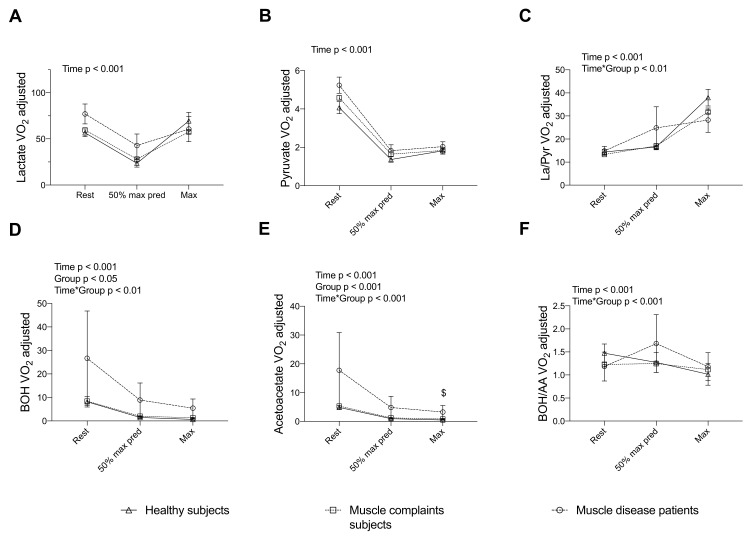
V’O_2_-adjusted biological parameters measured by routine automate in healthy subjects, muscle complaint subjects and muscle disease patients during CPET: (**A**) lactate; (**B**) pyruvate; (**C**) lactate/pyruvate ratio; (**D**) beta-hydroxybutyrate; (**E**) acetoacetate and (**F**) beta-hydroxybutyrate/acetoacetate ratio. Each patient was measured at three timepoints: rest, 50% of predicted maximal intensity and at maximal intensity. Data are presented as mean ± SEM at each time point. Significant *p*-value for post-hoc test $: healthy vs. muscle complaint subjects. AA: Acetoacetate; BOH: beta-hydroxybutyrate; CPET: Cardiopulmonary Exercise Test; La: lactate; Pyr: pyruvate; SEM: Standard Error of the Mean.

The V’O_2_-adjusted values of plasma La, Pyr, La/Pyr, BOH, AA and BOH/AA at rest, V_T1_ and V’O_2max_ from the three groups are reported in Figure 2. Time effects were significant for all parameters. Yet, because the marker was adjusted to the exercise intensity, there was a significant decrease in La, Pyr, BOH and AA between rest and maximum exercise (all *p* < 0.05). The La/Pyr ratio increased in all groups (*p* < 0.05), while BOH/AA ratio decreased in healthy subjects only (*p* < 0.05).

A significant group-effect for V’O_2_-adjusted BOH and AA (*p* < 0.005), with higher values in muscle disease patients vs. other groups, was reported. Significant Group*Time interactions could be observed for the La/Pyr ratio, BOH, AA and BOH/AA (*p* < 0.05), in line with a different time course of the exercise intensity-adjusted markers between groups. Regarding the BOH/AA ratio, a 31.1% decrease between rest and max exercise in healthy subjects while no difference (−0.3%) in muscle disease patients were observed. Post-hoc tests showed that only AA at V’O_2max_ was significantly increased in muscle disease patients compared to muscle complaint subjects.

In addition, when comparing healthy subjects with muscle complaint subjects classified according to their degree of exercise intolerance, the exercise adaptations of energy substrates in the most severe subjects (V’O_2max_ < 60% pred., *n* = 42) matched the blunted adaptations observed in muscle disease patients. Indeed, V’O_2_-adjusted La, Pyr, BOH and AA were significantly increased in this physically impaired and muscle complaint subgroup. In addition, a Group*Time interaction was found for both La/Pyr and BOH/AA ratios (Supplementary Figure S1).

### 3.4. Completing the Assessment of the TCA Cycle Intermediates during a CPET Reveals a Blunted Exercise-Induced Adaptation of Other Intermediates in Muscle Complaint Subjects with Severe Exercise Intolerance

**Figure 3:**
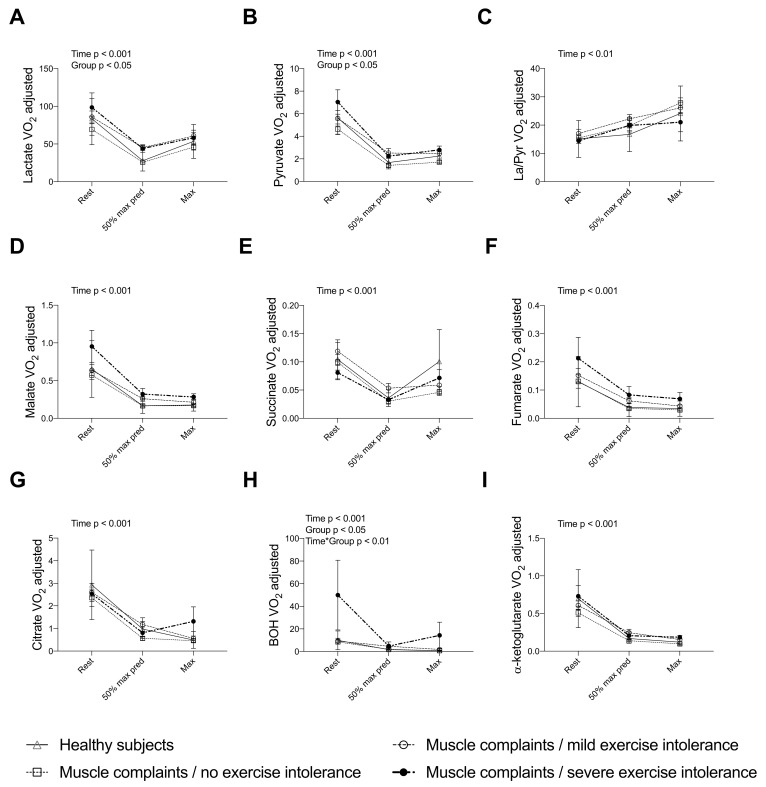
V’O_2_-adjusted TCA cycle intermediates measured by LC-MS/MS in healthy subjects, muscle complaint subjects with no (V’O_2max_ > 85% pred.), mild (60% pred. < V’O_2max_ < 85% pred.), and severe (V’O_2max_ < 60% pred.) exercise intolerance during CPET: (**A**) lactate; (**B**) pyruvate; (**C**) lactate/pyruvate ratio; (**D**) malate; (**E**) succinate; (**F**) fumarate; (**G**) citrate; (**H**) beta-hydroxybutyrate and (**I**) alpha-ketoglutarate. Each patient was measured at three timepoints: rest, 50% of predicted maximal intensity and at maximal intensity. Data are presented as mean ± SEM at each time point. BOH: beta-hydroxybutyrate; CPET: Cardiopulmonary Exercise Test; La: lactate; Pyr: pyruvate; SEM: Standard Error of the Mean; TCA: Tricarboxylic Acid.

Simultaneous determination of energy, redox markers and TCA intermediates for healthy (*n* = 10) and some of muscle complaint subjects (*n* = 39) confirmed our results above using the enzymatic routine method, i.e., the blunted adaptations of the V’O_2_-adjusted La, Pyr, BOH, AA and their ratios. Yet, no significant difference in the time course of V’O_2_-adjusted TCA cycle intermediates was found between healthy and muscle complaint subjects, whatever the degree of exercise intolerance (Figure 3).

## 4. Discussion

The V’O_2_-adjusted blood energy substrates and redox markers during a CPET showed significant correlations with muscle function and mitochondrial respiration. Thus, adding V’O_2_-adjusted blood biomarkers and ratios to muscle function parameters improved the determination of the V’O_2max_. Interestingly, no increase in La, Pyr, BOH and AA was observed when adjusted to the exercise intensity corresponding to the sampling time during the CPET. Yet, the time course of these biomarkers and their ratios were blunted in patients with muscle disease and in muscle complaint subjects with the most severe exercise capacity impairment.

### 4.1. Validity of Exercise Values of V’O_2_-Adjusted Energy Substrates and Muscle Function/Respiration

Physiologically, blood concentrations in energy metabolites change with exercise, revealing mobilization, utilization, and conversion of energy metabolites to meet the ATP demand of the exercising muscles. The plasma TCA intermediates and glycolysis products increase with short term exercise [17,19,25]. Indeed, the TCA cycle intermediate pool size increases via anaplerosis/cataplerosis imbalance [4,33]. However, previous studies showed that TCA cycle intermediates and the exercise intensity [19], as well as the maximal exercise capacity of a subject [17,21], were positively correlated, showing that the increases in TCA cycle intermediates during exercise is related to the relative intensity of muscle contraction (as a percentage of its maximal exercise capacity). The relative intensity dependence of the plasma TCA cycle intermediates constitutes a major bias for the diagnosis of a muscle disease. Indeed, for a given exercise, the relative intensity will be higher in a patient with muscle disease vs. a healthy subject, and will lead to increased plasma TCA cycle intermediates during exercise in muscle disease patients. Hammaren et al. tried to take into account this limit in the context of the “lactate stress test” in patients with mitochondrial myopathy [34]. The authors proposed to adjust the intensity of the lactate stress test to the reduced exercise capacity of the patient. This individualization of the test intensity allowed improvement of the diagnosis performance of the lactate values during exercise [34]. In our study, the exercise-intensity incrementation was also individualized to the subject, but we also reported the blood metabolites to the intensity (i.e., the V’O_2_ in % of the predicted V’O_2max_) at which the sampling has been obtained. Thus, the metabolite level was clearly adjusted to the relative exercise intensity, avoiding any “exercise intensity bias”. This exercise intensity adjustment allowed specific assessment of the muscle energy metabolism of the subject.

In our study, we observed a significant decrease in the V’O_2_-adjusted La and Pyr during exercise in any groups (time effect: *p* < 0.001 and *p* < 0.001, respectively). However, the intensity-adjusted La, Pyr, BOH, and AA were inversely correlated with functional parameters related to the muscle function (V’O_2_/W slope, V’O_2_@V_T1_, V’O_2max_, Table 1), meaning that the better the muscle capacity is, the fewer muscle energy metabolites are released. In addition, in multivariate analysis, adding V’O_2_-adjusted BOH (both at V_T1_ and at maximal exercise) and AA@max to the muscle function parameters increased the adjusted R^2^ (Table 2), meaning that the determination of the V’O_2max_ was improved by the assessment of the intensity-adjusted BOH or AA during exercise. This result confirms the independent correlations between BOH/AA at maximum and submaximal exercise intensities and V’O_2max_.

Furthermore, correlations between the intensity-adjusted metabolites during CPET and the mitochondrial respiration parameters were tested in a subgroup of subjects with muscle symptoms, but without muscle disease (*n* = 12). Previously published correlations between V_max_ glutamate and V’O_2max_ and V’O_2_@V_T1_ [15] could be corroborated here which confirm the validity of our oxygraphy measurements in permeabilized fibers. In this context, we observed statistically significant inverse correlations between V’O_2_-adjusted La, Pyr, at submaximal exercise and V_max_ pyruvate (Supplementary Table S1), and between V’O_2_-adjusted BOH and AA and V_max_ glutamate (Supplementary Table S1). While the lactate disposal during exercise is mainly dependent on the mitochondrial oxidation, the blood lactate removal during a supra-maximal exercise is also related to both V_max_ glutamate + malate and V_max_ pyruvate + lactate [18], in agreement with previous studies [15,35]. Altogether, the plasma lactate during exercise appears to be driven by the mitochondrial function. Indeed, deficits in respiratory chain complex also lead to an increased plasma BOH/AA ratio during exercise. Complex I deficit reduces V_max_ glutamate and V_max_ pyruvate. In addition, complex I inhibition induces an accumulation of mitochondrial but not cytosolic NADH, which leads to increases in the BOH/AA ratio [36], but not in the La/Pyr ratio [37]. Conversely, BOH plays a beneficial role on muscle mitochondrial oxidation [38] in permeabilized fiber [39], and on exercise capacity and mitochondrial function in vivo [40,41]. Combined with a 12-week exercise training program, a ketogenic diet in healthy subjects improved the mitochondrial respiratory function in permeabilized fibers [42].

Altogether, the V’O_2_-adjusted La, Pyr, BOH and AA at submaximal and maximal exercise intensities during exercise demonstrated their validity vs. functional and respiratory indexes of the skeletal muscle.

### 4.2. Discriminative Value of Exercise-Induced Increase in Lactate, Pyruvate and Ratios in Muscle Diseases

In our study, patients with muscle disease showed impaired functional exercise capacity markers and V’O_2max_ compared to healthy subjects, as previously reported [11,12,13,19]. Regarding energy substrate, we found no Group*Time interaction for La and Pyr, meaning that there was no blunted exercise adaptations of these metabolites during exercise, in contrast to previous studies in McArdle’s disease (blunted release) [43] and in mitochondrial myopathy patients (enhanced release) [34]. Yet, relative exercise intensity during the exercise work test should have been exaggerated in mitochondrial myopathy patients vs. healthy controls [34].

In our study, there was a significant Group*Time effect for the La/Pyr ratio during CPET. Yet, post-hoc tests did not show significant differences between groups, because of insufficient power. The 67% increase in the La/Pyr ratio at the estimated V_T1_ could be confirmed in a larger cohort. Indeed, during exercise, lactate and pyruvate are in equilibrium with a high NAD/NADH cytosolic ratio (low NADH) [22]. Thus, the altered cytosolic redox status during exercise is evidenced with an increase in La/Pyr ratio. While previous works showed a good specificity of the La/Pyr ratio in mitochondrial myopathies at rest [44], the discriminating value of the La/Pyr ratio was acceptable only when the lactate level was elevated [45,46,47]. Thus, given that an increase in La during exercise was found, the La/Pyr ratio may be discriminative in muscle diseases in our study. In addition, the Mitochondrial Medicine Society recommends the assessment of blood or cerebrospinal fluid La/Pyr ratio in the diagnosis strategy of mitochondrial disease patients [48]. Our study allows to extend this recommendation to the assessment of La/Pyr ratio during a CPET. Indeed, an international consensus recommends aerobic exercise training in mitochondrial disease patients [49,50], which requires a pre-training CPET. Thus, extending the recommendation to assess La, Pyr and La/Pyr ratio during a CPET would provide the most discriminative (i.e., functional and intensity-adjusted) markers and foster exercise training prescription for subjects with muscle function impairment.

### 4.3. Discriminative Value of Exercise-Induced Increase in β-Hydroxybutyrate, Acetoacetate and Ratio in Muscle Diseases

In our study, intensity-adjusted BOH and AA showed a significant Group effect. If a significant post-hoc test was found only for AA at maximum exercise (probably due to insufficient statistical power), it indicates an abnormal ketogenesis or an impairment of the lipid metabolism in muscle disease patients. β-hydroxybutyrate and AA are ketone bodies responsible for the energy transport from the liver to other tissues [51]. The activation of the lipid energy metabolism, during prolonged muscle exercise in particular, leads to a production of BOH and AA [52], and contributes to a physiological ketosis. Plasma BOH was systematically increased after exercise in five studies, while plasma AA increase after exercise was more equivocal [25]. Yet, in our muscle disease patients, the adjusted BOH and AA levels were in line with the increased raw levels reported in several pathological conditions (such as diabetes, intoxication or inherited metabolism diseases [53]). In addition, BOH and AA are in equilibrium with a low NAD/NADH ratio (high NADH) in the mitochondria. Thus BOH/AA ratio reflects the NAD/NADH ratio in the mitochondria. In our study, significant Time (*p* < 0.001) and Group*Time interaction (*p* < 0.001) effects were observed for the V’O_2_-adjusted BOH/V’O_2_-adjusted AA ratio, meaning a blunted adaptation of the mitochondrial NAD/NADH ratio during exercise in muscle disease patients. Interestingly, no significant increase in the BOH/AA ratio occurred in healthy subjects, in line with two previous exercise studies in healthy subjects [23,54]. While an abnormal elevation of the BOH/AA ratio has been related to different disorders (TCA cycle, mitochondrial respiratory chain, ketogenesis or ketolysis [55]), the blunted adaptations of both La/Pyr and BOH/AA ratios during CPET in muscle disease patients argue for a disorder of the muscle fiber redox state during exercise (elevated NADH/NAD ratio or NADH reductive stress) [37,56].

### 4.4. Screening for “At-Risk” Patients for Muscle Oxidative Metabolism Diseases

While genetic diagnosis performance is currently improving, it usually requires an expert center with cost-expensive analyses and expertise. Moreover, in many mitochondrial or inherited muscle diseases, genetic mutations are not pathognomonic, such as the m.3245A>G mutation in the MT-TL1 gene in MELAS (found in 80% of patients), and not systematically associated with the pathology while being carried by 1/400 white Europeans [57,58]. Therefore, muscular biopsy [59] and a non-invasive biomarker with good positive and negative predictive values is still highly warranted for clinical practice. Currently, the assessment of a full metabolomic profile has shown promise for the diagnosis of muscle diseases [60]. New metabolite exercise-induced adaptations in healthy subjects have been identified [17,19]. Explorations of blood metabolite adaptations to exercise could substitute to the invasive muscle biopsy-assessment, since the plasma increase in TCA cycle intermediates during exercise mirrors the TCA cycle expansion in the muscle (succinate, malate, and fumarate) [17,33]. Plasma assessments at peak exercise in patients with MELAS revealed an abnormal TCA cycle intermediate profile, with increases in citrate, aconitate, isocitrate, and malate, and a decrease in succinate [61].

In our study, although we were unable to complete the panel assessment of TCA cycle intermediates in muscle disease patients, we were able to complete that of the muscle complaint group. In this group (*n* = 230), reductions in their V’O_2max_ and peak lactate were in line with data reported in a previous study including symptomatic subjects, with no evidence of muscle disease in the muscle biopsy [62]. Of note, a muscle biopsy was performed in 12 subjects in our muscle complaint group and did not reveal evidence of a muscle disease. In our muscle complaint group, it is striking to note that subjects with the poorest exercise capacity (V’O_2max_ < 60% pred.) showed increased plasma V’O_2_-adjusted La, Pyr, BOH, AA and ratios during exercise that matched the blunted adaptations observed in muscle disease patients. Thus, in this subgroup, it is possible that the muscle biopsy could have revealed a muscle disease. Indeed, in a similar cohort of 113 explored subjects between 2005 and 2010, a retrospective analysis showed a prevalence of biopsy-proven muscle disease of 15% (unpublished data). Identifying the key-exercise metabolites with a high sensibility that would eliminate the probability of a negative muscle biopsy appears as a relevant strategy when the pre-test probability of muscle disease is low. Altogether, further studies including a larger number of subjects with muscular symptoms and available muscle biopsy are required to establish a definitive blood profile identifying high risk subjects to muscle disease.

### 4.5. Study Limitations

The medical examination and the cardiopulmonary exercise test showed no evidence of medical disease in the subjects of the control group. Thus, these subjects can be considered as healthy. Yet, from a muscle perspective, no biopsy was performed to eliminate any muscle disease in these healthy subjects. In addition, the muscle disease patients’ group was constituted by patients with different diagnoses that could increase its heterogeneity. Given the statistically significant differences between groups reported in our study, the heterogeneity in the muscle disease group was not enough to mask the differences in muscle functional and plasma parameters between the study groups. Yet, because our “muscle disease patient group” was mostly constituted by metabolic myopathies, the discriminative value of the intensity adjusted plasma biomarkers during exercise should be assessed in patients with other muscle diseases.

### 4.6. Exercise Metabolomics: Potential Issues for Nutritional Interventions

Besides diagnosis issues in muscle diseases, improving the assessment of the muscle metabolic adaptations to exercise in subjects with muscle complaints or exercise intolerance has an obvious relevance in nutrition. While treatment remains challenging in this context, nutritional strategies constitute the first line treatment in inherited muscle diseases [63]. In McArdle disease, studies have emphasized the efficacy of regular exercise associated with a carbohydrate-rich diet, pre-exercise sucrose or creatine [64]. In carnitine palmitoyltransferase II (CPTII) deficiency, a carbohydrate-rich diet associated with a reduction in dietary fat is classically proposed. Recently, a dietary alternative with carbohydrate reduction and supplementation in triheptanoin showed a potential to prevent rhabdomyolysis and exercise intolerance [65].

Currently, the nutritional supplementation in subjects with muscle symptoms of exercise intolerance is at its very beginning. Nutritional supplements improving muscle lipid metabolism (carnitine or medium chain triglycerides), or boosting muscle bioenergetics (creatine monohydrate, proteins/EAAs, or Vitamin D), or reducing exercise-induced muscle damage and related inflammation (Omega3 fatty acids, Polyphenols [66]) could be beneficial in selected patients. The hypothesis of a clinical benefit of a nutritional supplementation in metabolic myopathies is suggested by evidence for nutritional supplement effects (curcumin [67], ginger [68]) in pain management in metabolic myopathies. The simultaneous assessment of metabolites in many energy pathways has allowed the identification of specific metabolomic signatures in muscle diseases [61], in professional athletes and in control subjects [21]. All in all, based on the assessment of metabolic biomarker adaptations to exercise, nutritional supplementations could be targeted in subjects with undiagnosed muscle disease but with muscle complaints or exercise capacity impairment. Personalization of the nutritional supplementation [69] based on the exercise metabolomics constitutes a promising issue to prevent rhabdomyolysis and muscle function decline.

## 5. Conclusions

Currently, the diagnosis strategy in muscle diseases with oxidative muscle impairment involves a combination of methods. The combination of functional parameters obtained during an exercise test and blood energy substrate levels (La, Pyr) and the redox balance markers (La/Pyr and BOH/AA ratios) approach reveals blunted adaptations during exercise in muscle disease patients. When adjusted for relative exercise intensity, La and Pyr constitute valid markers, showing good correlations with muscle function and respiration indexes. In addition, La/Pyr and BOH/AA ratios during exercise enabled discrimination of healthy subjects with patients with muscle disease, in line with the disturbed redox state in the exercising muscle of muscle disease patients. Last, the subgroup of muscle complaint subjects having the poorest exercise capacity which is “at-risk” of muscle disease showed similar blunted adaptations of studied biomarkers and their ratios. This new metabolomics approach has potential in the diagnosis strategy to further improve the diagnosis probability and indications of muscle biopsies in subjects with muscle complaints, as well as to target personalized nutritional supplementations.

## Figures and Tables

**Table 1:** Univariate Pearson’s correlation of physiologic exercise parameters with plasma biological parameters measured at each time point (@rest: resting value; @eV_T1_: at estimated first ventilatory threshold; @max: at the acme of effort) in healthy and muscle complaint subjects (*n* = 240). AA: acetoacetate; BOH: β-hydroxybutyrate; La: lactate; Pyr: pyruvate; V’O_2_/W slope: slope of V’O_2_/work rate relationship change; V’O_2_@V_T1_: V’O_2_ at first ventilatory threshold; V’O_2max_: V’O_2_ at the maximal effort. * *p*-value < 0.05; ** *p*-value < 0.01; *** *p*-value < 0.001).

Variables	Weariness	V’O_2_/W Slope	V’O_2_@V_T1_	V’O_2max_
La@rest	−0.021	0.048	−0.242 ***	−0.235 ***
La@eV_T1_	0.002	−0.142	−0.582 ***	−0.566 ***
La@max	−0.023	−0.221 **	−0.306 ***	−0.384 ***
Pyr@rest	0.028	−0.010	−0.324 ***	−0.313 ***
Pyr@eV_T1_	0.048	−0.101	−0.663 ***	−0.613 ***
Pyr@max	0.136	−0.333 ***	−0.492 ***	−0.650 ***
La/Pyr@rest	−0.054	0.071	0.074	0.028
La/Pyr@eV_T1_	−0.072	−0.117	−0.166 *	−0.222 **
La/Pyr@max	−0.185 *	0.020	0.192 **	0.276 ***
BOH@rest	−0.009	−0.098	−0.170 *	−0.196 **
BOH@eV_T1_	−0.055	−0.073	−0.258 ***	−0.251 ***
BOH@max	−0.027	−0.113	−0.168 *	−0.224 **
AA@rest	0.067	−0.065	−0.174 **	−0.197 **
AA@eV_T1_	0.042	−0.081	−0.350 ***	−0.324 ***
AA@max	0.078	−0.122	−0.194 **	−0.277 ***
BOH/AA@rest	−0.018	−0.156	−0.032	−0.096
BOH/AA@eV_T1_	−0.078	−0.058	−0.010	−0.071
BOH/AA@max	−0.053	−0.128	−0.024	−0.092

**Table 2:** Multivariate model analysis using functional exercise parameters and V’O_2_-adjusted biological parameters in healthy and muscle complaint subjects (*n* = 240) to explain V’O_2max_. (@rest: resting value; @V_T1_: at first ventilatory threshold; @max: at the acme of effort), AA: acetoacetate; BOH: β-hydroxybutyrate; La: lactate; Pyr: pyruvate; V’O_2_/W slope: slope of V’O_2_/work rate relationship change; V’O_2_@V_T1_: V’O_2_ at first ventilatory threshold; V’O_2max_: V’O_2_ at the maximal effort. R^2^: model determination coefficient; R^2^ adjusted: model determination coefficient adjusted to the number of variables; Mallow’s Cp: Cp coefficient of Mallow.

Nr. of Variables	Variables	R^2^	R^2^ Adjusted	Mallow’s Cp
1	V’O_2_@V_T1_	0.527	0.521	44.62
2	V’O_2_/W slope + V’O_2_@V_T1_	0.619	0608	23.85
3	V’O_2_/Wslope + V’O_2_@V_T1_ + AA@max	0.641	0.627	20.20
4	V’O_2_/Wslope + V’O_2_@V_T1_ + BOH@eV_T1_ + BOH@max	0.681	0.663	12.42
5	V’O_2_/Wslope + V’O_2_@V_T1_ + Pyr@max + BOH@eV_T1_ + BOH@max	0.706	0.685	8.21
6	V’O_2_/Wslope + V’O_2_@V_T1_ + La@max + La/Pyr@rest + La/Pyr@eV_T1_ + La/Pyr@max	0.723	0.699	5.83
7	V’O_2_/Wslope + V’O_2_@V_T1_+ La@rest + La@max + Pyr@rest + La/Pyr@eV_T1_ + La/Pyr@max	0.735	0.708	4.82
8	V’O_2_/Wslope + V’O_2_@V_T1_ + La@max/ + La/Pyr@rest + La/Pyr@eV_T1_ + La/Pyr@max + AA@eV_T1 +_ AA@max	0.750	0.721	3.08

**Table 3:** Characteristics of healthy subjects, muscle complaint subjects and muscle disease patients.

	Healthy Subjects (*n* = 10)	Muscle Complaint Subjects (*n* = 230)	Patients with Muscle Disease (*n* = 9)	*p*-Value
Sex female (n; %)	5; 50	118; 51.3	4; 44.4	1.000
Age	37.1 ± 13.3	44.9 ± 15.8	36.3 ± 16.0	0.115
BMI kg/m^2^	22.7 ± 2.3	24.9 ± 5.0	24.9 ± 3.2	0.343
Lean mass (%)	51.9 ± 9.2	50.6 ± 11.4	50.2 ± 13.4	0.896
Skeletal Muscle Index (kg/m^2^)	8.4 ± 1.4	8.8 ± 2.6	8.7 ± 2.2	0.909
V’O_2max_% predicted	100.7 ± 17.0	82.8 ± 23.6	62.2 ± 22.6 ^£^	0.0008
V’O_2_@V_T1_% V’O_2max_	65.7 ± 17.4	57.0 ± 16.3	51.3 ± 19.9	0.131
Weariness (/10)	0 ± 0	3.9 ± 2.8 ^$^	5.6 ± 2.8 ^£^	<0.0001
V’O_2_/work rate slope (mL/min/Watt)	8.7 ± 1.2	8.8 ± 2.0	8.1 ± 0.4	0.577

The *p*-value was measured using one-way analysis of variance Kruskal–Wallis test; when variance was significantly different, Dunn’s post hoc test was realized with *p*-value < 0.05 as significant. $: healthy vs. muscle complaint; £: healthy vs. muscle disease.

## Data Availability

No new data were created or analyzed in this study. Data sharing is not applicable to this article.

## Supplementary Materials

The following supporting information can be downloaded at: https://www.mdpi.com/article/10.3390/nu14091886/s1, Figure S1: V’O_2_-adjusted lactate, pyruvate, Beta-hydroxybutyrate, acetoacetate and La/Pyr and BOH/AA ratios in healthy subjects, symptomatic subjects with no, mild (V’O_2max_ > 85% pred.), moderate (V’O_2max_ < 85% pred.), and severe (V’O_2max_ < 60% pred.) exercise intolerance during CPET. Data are presented as mean ± SEM at each timepoints. *p*-value significative for post hoc test healthy vs symptomatic $; healthy vs muscle disease £; Table S1: Univariate Pearson’s correlation of muscle physiologic parameters and V’O_2_-adjusted energy substrates parameters measured at each timepoints in the healthy and symptomatic groups (*n* = 240). * *p*-value < 0.05; ** *p*-value < 0.01; *** *p*-value < 0.001.
