# Supplementary material for: Biomarkers of Redox Balance Adjusted to Exercise Intensity as a Useful Tool to Identify Patients at Risk of Muscle Disease through Exercise Test
Source: Nutrients. 2022 Apr 29;14(9):1886. doi: 10.3390/nu14091886 (PMC9105000; doi:10.3390/nu14091886)

Supplementary material:

Plasma muscle metabolites during a maximal exercise test can discriminates subjects with muscle symptoms and disease

Methods

Muscle biopsy was immediately placed in an ice-cold relaxing solution [at ionic strength 160 (potassium methanesulfonate), pH 7.1] containing (in mM) 10 EGTA-calcium buffer (free Ca2concentration: 100 nmol/l), 20 imidazole, 3 KH2PO4, 1 MgCl2, 20 taurine, 0.5 DTT, 5 MgATP, and 15 phosphocreatine. The fiber bundles were separated with sharp-ended needles, leaving only small areas of contact, and were incubated in 1 ml of the above solution (4°C) containing 50 g/ml saponin for 30 min with continuous stirring. To completely remove the saponin, the fibers were washed with continuous stirring with relaxing solution for 10 min (4°C); to remove free ATP, they were then washed with oxygraph solution for 2 5 min (4°C). This was of the same composition as the relaxing solution, except that MgATP and phosphocreatine were replaced by 2 mM malate, 3 mM phosphate, and 2 mM fatty acid-free bovine serum albumin (pH 7.1). After washing, the fibers were stored on ice in oxygraph solution until determination of mitochondrial respiration activity.

The respiratory parameters of the total mitochondrial population were studied in situ, as previously described (25, 37), Measurements were carried out at 30°C with continuous stirring in 3 ml of the oxygraph solution with different respiratory substrates (in mM):

- Glutamate (5 mM)/malate (2 mM)
- Pyruvate (10 mM)/malate (2 mM)

ADP-stimulated respiration above basal oxygen consumption was measured by stepwise addition of ADP (2.5–2,000 μM). Glutamate and malate are substrates of complex I, and allow activation of complexes I, III, and IV of respiratory chain. Pyruvate feeds the TCA cycle (via Acetyl-CoA) After the following respiratory measurements, the fiber bundles were removed, dried overnight, and weighed the next day. Respiration rates were expressed in micromoles of O2 per minute per gram of dry weight. Maximal ADP-stimulated respiration (V'max) for each substrate was calculated by using a nonlinear monoexponential fitting of the Michaelis-Menten equation with DataFit 6.0 software. The acceptor control ratio was calculated as V'max/V'0 oxygen consumption.

Results

Table S1. Univariate Pearson's correlation of muscle physiologic parameters and $V'O_2$-adjusted energy substrates parameters measured at each timepoints in the healthy and symptomatic groups (n=240). *p-value <0,05 ; **p-value <0,01 ; ***p-value <0,001.

Variables	glutamate Vo	Glutamate Vmax	pyruvate Vo	pyruvate Vmax	palmitoyl CoA Vo	palmitoyl CoA Vmax
**Weariness**	-0,214	-0,235	-0,290	-0,357	**-0,800***	-0,642
**$V'O_2$/work rate slope**	-0,870	-0,995	-0,219	0,369	-0,213	-0,987
**$V'O_2@V_{T1}$**	0,596	**0,640***	**0,660***	**0,688***	0,611	**0,687***
**$V'O_{2max}$**	0,590	**0,739****	**0,732****	**0,714****	0,614	**0,698***
**La@rest**	-0,250	-0,092	-0,199	-0,322	-0,189	-0,066
**La@eV_{T1}**	-0,426	-0,424	-0,373	**-0,684***	-0,351	-0,441
**La@max**	**-0,603***	-0,188	-0,562	-0,400	-0,578	-0,361
**Pyr@rest**	-0,279	-0,082	-0,226	-0,449	-0,174	-0,027
**Pyr@eV_{T1}**	-0,430	-0,376	-0,395	**-0,649***	-0,322	-0,400
**Pyr@max**	-0,558	-0,392	**-0,607***	-0,567	-0,412	-0,365
**La/Pyr@rest**	-0,083	-0,172	-0,127	0,061	-0,275	-0,363
**La/Pyr@eV_{T1}**	0,173	-0,022	0,168	0,083	0,027	-0,015
**La/Pyr@max**	-0,233	0,217	-0,103	0,181	-0,362	-0,098
**BOH@rest**	-0,385	**-0,729***	-0,530	-0,599	-0,356	-0,658
**BOH@eV_{T1}**	-0,245	**-0,765***	-0,488	-0,487	-0,238	-0,630
**BOH@max**	-0,194	-0,748	-0,465	-0,461	-0,193	-0,572
**AA@rest**	-0,392	-0,442	-0,379	-0,368	-0,479	-0,474
**AA@eV_{T1}**	-0,107	**-0,608***	-0,392	-0,325	-0,185	-0,447

**AA@max**	-0,125	**-0,621***	-0,426	-0,294	-0,221	-0,449
**BOH/AA@rest**	-0,334	**-0,744***	-0,403	-0,286	-0,288	-0,671
**BOH/AA@eV$_{T1}$**	-0,299	-0,688	-0,440	-0,328	-0,262	-0,649
**BOH/AA@max**	-0,164	-0,736	-0,328	-0,484	-0,060	-0,536

Figure S1. V’O_2-adjusted lactate, pyruvate, B-hydroxybutyrate, acetoacetate and La/Pyr and BOH/AA ratios in healthy subjects, symptomatic subjects with no, mild (V’O_{2max}>85%pred.), moderate (V’O_{2max}<85%pred.), and severe (V’O_{2max}<60%pred.) exercise intolerance during CPET. Data are presented as mean ± SEM at each timepoints. *p*-value significative for post hoc test healthy vs symptomatic $; healthy vs muscle disease £.

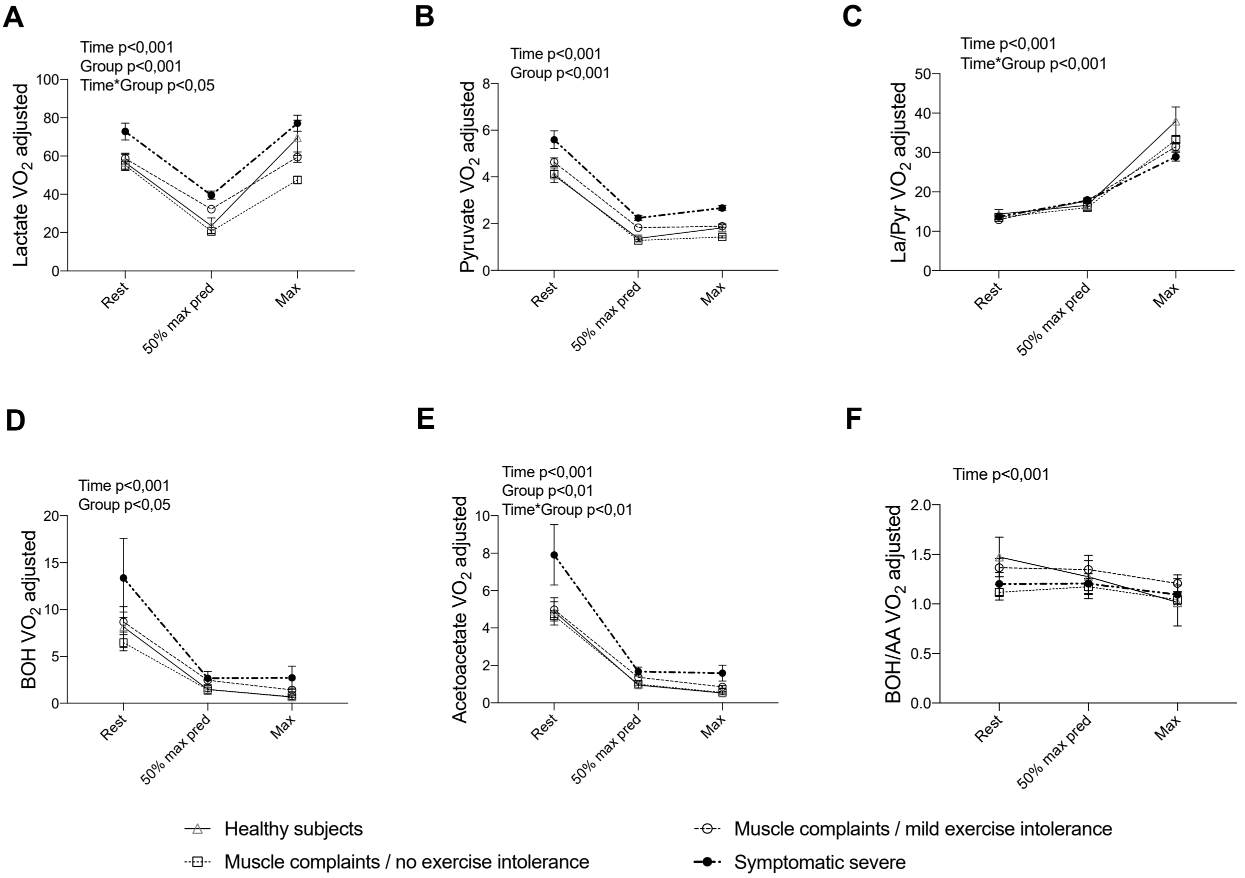

Supplement: Supplementary file 1 [file nutrients-14-01886-s001.zip › nutrients-1668326-supplementary.pdf]
